# Enhancing plasmonic superconductivity in layered materials via dynamical Coulomb engineering

**DOI:** 10.1038/s41699-026-00668-3

**Published:** 2026-01-28

**Authors:** Y. in ’t Veld, M. I. Katsnelson, A. J. Millis, M. Rösner

**Affiliations:** 1 I. Institute of Theoretical Physics, Universität Hamburg, Hamburg, Germany; 2 Institute for Molecules and Materials, Radboud University, Nijmegen, The Netherlands; 3 Constructor Knowledge Institute, Constructor University, Bremen, Germany; 4 Center for Computational Quantum Physics, Flatiron Institute, New York, NY USA; 5 Department of Physics, Columbia University, New York, NY USA; 6 Faculty of Physics, Bielefeld University, Bielefeld, Germany

**Keywords:** Materials science, Nanoscience and technology, Optics and photonics, Physics

## Abstract

Conventional Coulomb engineering, through controlled manipulation of the environment, offers an effective route to tune the correlation properties of atomically thin van der Waals materials via static screening. Here we present tunable *dynamical* screening as a method for precisely tailoring bosonic modes to optimize many-body properties. We show that “bosonic engineering” of plasmon modes can be used to enhance plasmon-induced superconducting critical temperatures of layered superconductors in metallic environments by up to an order of magnitude, due to the formation of interlayer hybridized plasmon modes with enhanced superconducting pairing strength. We determine optimal properties of the screening environment to maximize critical temperatures. We show how bosonic engineering can aid the search for experimental verification of plasmon mediated superconductivity.

## Introduction

"Coulomb engineering”^1,2^ aims to tailor many-body effects via modifications to the fundamental electron-electron interactions. Atomically thin van-der-Waals materials are promising candidates to exploit this, because the low dimensionality enhances correlation effects while the large surface-to-volume ratio allows for efficient external manipulation. As a result, when van-der-Waals materials are placed on substrates or when they are encapsulated by other materials, the modified environmental screening properties can modify the range^3,4^, strength^2^ and dynamical behavior^1^ of the Coulomb interaction.

The environmental modification of screened dynamical interactions presents an intriguing many-body engineering route characterized by a tailored interplay of internal and external bosonic modes. Examples include plasmons or phonons in the environment coupling to the Fermi sea of a layered van-der-Waals metal leaving behind additional spectral features^5^ or substrate phonons acting on excitons in 2D semiconductors^6,7^. Depending on the degree of coupling, these modes can either act individually or in a composite manner to affect the electronic properties of the two-dimensional (2D) material, opening up the possibility for “bosonic engineering”: i.e. the control of dynamical interactions by external means. Substrate phonon enhancement of electron pairing has been proposed for superconducting monolayers^8–14^ and hybridized composite bosons such as plasmonic multicomponent modes or interacting plasmon-phonon modes were discussed in particular with respect to their effects on superconductivity in single-layer^15^ as well as stacked^16–20^ and/or twisted layered materials^21–23^.

Here we take these ideas one step further, showing how bosonic engineering via dynamical external screening of the Coulomb interaction can drastically enhance plasmon-induced superconducting critical temperatures in layered materials by strengthening the plasmon-induced superconducting pairing at low frequencies, without also increasing the high-frequency Coulomb-induced repulsion.

## Results

**Fig. 1:**
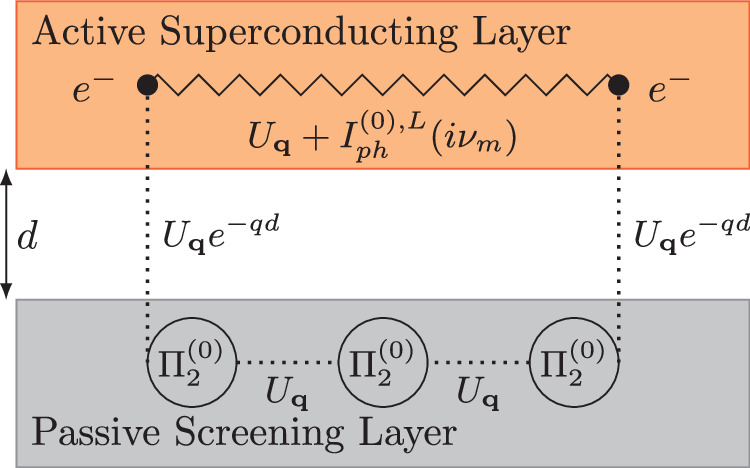
Schematic illustration of the studied heterostructure. The top orange layer is the active layer, which hosts superconductivity. The bottom gray layer is a screening layer which dynamically screens the active layer via the long-range Coulomb interaction. The dotted lines represent (interlayer) bare Coulomb interactions, the circles represent polarization processes within the screening layer and the jagged line represents the bare (longitudinal) interactions within the active layer.

We study the hetero-bilayer system shown schematically in Fig. 1: an active superconducting layer, coupled via long-range Coulomb interactions to a passive metallic screening layer. We assume that the layers are electronically decoupled with no hybridization between the electronic wave functions in the two layers. This setup can for example be realized by two van-der-Waals materials separated by an insulating spacer, such as a monolayer of hexagonal Boron Nitride (hBN).

### Theoretical description

We describe the bilayer with the following Hamiltonian

1 $$\begin{array}{l}H=\mathop{\sum }\limits_{{\bf{k}}i\sigma }{\varepsilon }_{{\bf{k}},i}{c}_{{\bf{k}}i}^{\sigma ,\dagger }{c}_{{\bf{k}}i}^{\sigma }+\frac{1}{2}\mathop{\sum }\limits_{{{\bf{kk}}}^{{\prime} }{\bf{q}}}\mathop{\sum }\limits_{ij\sigma {\sigma }^{{\prime} }}{\widehat{U}}_{ij}({\bf{q}}){c}_{{\bf{k}}+{\bf{q}}i}^{\sigma ,\dagger }{c}_{{{\bf{k}}}^{{\prime} }-{\bf{q}}\,j}^{{\sigma }^{{\prime} },\dagger }{c}_{{{\bf{k}}}^{{\prime} }j}^{{\sigma }^{{\prime} }}{c}_{{\bf{k}}i}^{\sigma }\\ \,\,\,+\mathop{\sum }\limits_{{\bf{q}}\nu }{\omega }_{e}{b}_{{\bf{q}}\nu }^{\dagger }{b}_{{\bf{q}}\nu }+\mathop{\sum }\limits_{{\bf{kq}}\nu \sigma }g({b}_{{\bf{q}}\nu }+{b}_{-{\bf{q}}\nu }^{\dagger }){c}_{{\bf{k}}+{\bf{q}}1}^{\sigma ,\dagger }{c}_{{\bf{k}}1}^{\sigma }\end{array}$$

where ${c}_{{\bf{k}}i\sigma }^{\sigma ,(\dagger )}$ and ${b}_{{\bf{q}}\nu }^{(\dagger )}$ are the usual electronic and phononic annihilation (creation) operators, respectively, for layer *i*, spin *σ* and phonon mode *ν*. We assume that each layer has a single band *ε*_**k,***i*_ of free electrons and a Coulomb interaction *U*_**q**_. The active layer (*i* = 1) furthermore hosts longitudinal optical (LO) and transversal optical (TO) phonon modes, with electron-phonon coupling constants *g* and phonon energies *ω*_*e*_ which are assumed to be momentum-independent for simplicity. The passive layer (*i* = 2, assumed metallic) hosts a plasmon mode that is coupled to density fluctuations in the active layer via the long-range Coulomb interaction. In this situation the phonons and Coulomb interaction combine to yield respective effective LO and TO interactions ${I}_{ph}^{(0),LO}(i{\nu }_{m})$ and ${I}_{ph}^{(0),TO}(i{\nu }_{m})$ between the electrons in the same layer, which are accompanied by non-local Coulomb interactions within and between both layers. In this setup we can integrate out the electronic degrees of freedom of the passive layer, leaving behind a longitudinal interaction of the form

2 $${\widetilde{I}}_{1}^{(0),L}({\bf{q}},i{\nu }_{m})={U}_{{\bf{q}}}+{I}_{ph}^{(0),LO}(i{\nu }_{m})+{U}_{{\bf{q}}}{e}^{-qd}\frac{{\Pi }_{2}^{(0)}({\bf{q}},i{\nu }_{m})}{1-{U}_{{\bf{q}}}{\Pi }_{2}^{(0)}({\bf{q}},i{\nu }_{m})}{U}_{{\bf{q}}}{e}^{-qd}$$

with *U*_**q**_ = 2*πe*^2^/(*Aεq*), ${I}_{ph}^{(0),LO/TO}(i{\nu }_{m})=2{\omega }_{e}{g}^{2}/({(i{\nu }_{m})}^{2}-{\omega }_{e}^{2})$ and the polarization in the passive layer ${\Pi }_{2}^{(0)}({\bf{q}},i{\nu }_{m})$, *A* is the unit-cell area, *d* the distance between the two layers and *ε* is a global dielectric constant. In Fig. 1 we show schematically the different terms of ${\widetilde{I}}_{1}^{(0),L}({\bf{q}},i{\nu }_{m})$ within the layer from which they originate. The fully screened interaction is finally given by ${I}_{1}({\bf{q}},i{\nu }_{m})={I}_{1}^{L}({\bf{q}},i{\nu }_{m})+{I}_{ph}^{(0),TO}(i{\nu }_{m})$, with ${I}_{1}^{L}({\bf{q}},i{\nu }_{m})$ obtained from screening ${\widetilde{I}}_{1}^{(0),L}({\bf{q}},i{\nu }_{m})$ within the random phase approximation using the polarization in the superconducting layer ${\Pi }_{1}^{(0)}({\bf{q}},i{\nu }_{m})$. Further details including the derivation and detailed specification of the model can be found in the Methods section.

In the high-frequency limit *I*_1_(**q**, *iν*_*m*_ → *∞*) = *U*_**q**_ is simply the bare Coulomb repulsion in the superconducting layer, which is unaffected by screening from the passive layer. The passive screening layer thus tunes only the low-energy part of the interaction, without altering the high-energy repulsive part.

Bosonic engineering occurs here via tuning material properties of the passive layer and the distance between the passive and active layers to optimize superconductivity in the active layer. We use this model within a fully retarded and non-local one-loop Eliashberg solver as applied in ref. ^15^, to investigate how the distance *d* as well as the electronic properties (effective mass and the doping level) of the passive metallic layer control the superconducting properties of the active layer.

### Tunable composite bosonic modes

**Fig. 2:**
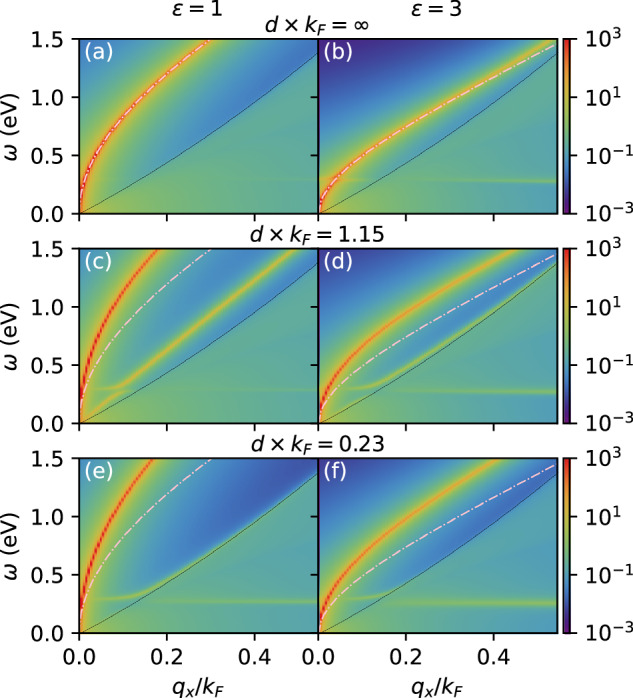
The effective interaction within the active layer. We show $2{\alpha }^{2}{F}_{{\bf{q}}}^{L}(\omega )/\omega$ for interlayer distances *d × k*_*F*_ = ∞ (**a**, **b**), *d × k*_*F*_ = 1.15 (**c**, **d**) and *d × k*_*F*_ = 0.23 (**e**, **f**) at *ε* = 1 (left panels) and *ε* = 3 (right panels). The dashed black lines denote the upper edges of the electron-hole continua and the dashed pink lines denote the plasmon dispersion of an isolated layer (i.e., at *d* × *k*_*F*_ = *∞*). These results were obtained at *T* = 100 K, with m* = 0.2*m*_*e*_ and *E*_*F*_ = 1 eV in both layers.

We start by investigating how bosonic Coulomb engineering affects ${\alpha }^{2}{F}_{{\bf{q}}}^{L}(\omega )=-{N}_{0}Im{I}_{1}^{L}({\bf{q}},\omega )/\pi$, the spectral function characterizing the longitudinal pairing modes in the active layer. Figure 2 depicts $2{\alpha }^{2}{F}_{{\bf{q}}}^{L}(\omega )/\omega$ for various interlayer distances *d* and environmental dielectric constants *ε*. For infinite layer distance [panels (a), (b)] the passive layer is irrelevant and we reproduce the hybridized phonon-plasmon mode of a monolayer in a static dielectric environment^15^. In this case, at *ε* = 3 there is hybridization between the dispersionless phonon mode and the $\sqrt{q}$-like two-dimensional plasmon mode, whereas at *ε* = 1 the Coulomb interaction screens out the phonon-mode, such that the phonon-plasmon hybridization is negligible. As the interlayer distance is decreased to *d* × *k*_*F*_ = 1.15 at *ε* = 1 [panel (c)], two distinct inter-layer plasmon modes appear, as is typical of coupled two-dimensional plasmon modes^24–29^. The high-energy mode can be understood as an in-phase oscillation of the total charge in both layers, which still has a $\sqrt{q}$-like dispersion in the long-wavelength limit, but shifted to higher energies compared to the plasmon mode at *d* = *∞* (indicated by the dashed pink line). The low-energy mode can be described as an out-of-phase dipolar oscillation between the charge densities in the two layers. It has a linear dispersion and lies below the energy of the isolated monolayer plasmon mode. As the interlayer distance decreases further to *d* × *k*_*F*_ = 0.23 [panel (e)], the out-of-phase mode shifts into the electron-hole continuum (boundary indicated by the dashed black lines), such that it is Landau damped if the layers are close enough. Also the LO phonon appears as an evident absorption feature at larger momenta.

Similar trends can be observed for *ε* = 3 [panels (d), (f)], but in this case the distance at which the out-of-phase mode starts to be Landau damped is larger. As for the longitudinal phonon mode, its coupling strength to the electrons (reflected by its intensity in Fig. 2) is not significantly affected by the dynamical interlayer screening. This is a consequence of the different energy scales of the phonon and plasmon modes. It does, however, hybridize more strongly with the dipolar plasmon mode than with the charged plasmon mode.

This shows that the individual bosons from the passive and active layers interact and hybridize in conceptually simple but quantitatively non-trivial ways, forming composite excitations with tunable dispersions and coupling strengths.

### Normal-state footprints

**Fig. 3:**
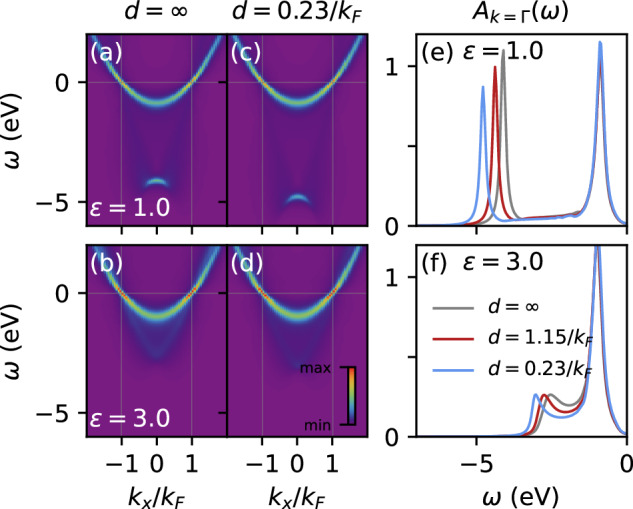
Momentum-resolved G_0_W_0_ spectral function. The spectral function *A*_**k**_(*ω*) within the active layer for different *ε* at *d* × *k*_*F*_ = *∞* (**a**, **b**) and *d* × *k*_*F*_ = 0.23 (**c**, **d**). **e**, **f** Linecut of the spectral function *A*_**k**_(*ω*) at **k** = *Γ* at *ε* = 1 and *ε* = 3. These results were obtained at *T* = 100 K, with m* = 0.2*m*_*e*_ and *E*_*F*_ = 1 eV in both layers.

In Fig. 3 we show the dressed electronic spectral function *A*_**k**_(*ω*) of the active layer in the G_0_W_0_ approximation. For infinite layer distance at *ε* = 3 [panel (b)] we find the typical phononic mass-enhancement around the Fermi energy, which is suppressed for *ε* = 1 [panel (a)] due to the screening by the Coulomb interaction. Below the band minimum we find additional spectral weight coming from plasmon polaron excitations. For *ε* = 1 these excitations induce a relatively coherent replica band, whereas for *ε* = 3 they induce an incoherent shoulder. The energy separation between the replica feature and the band minimum is determined by a representative energy scale of the plasmon dispersion^5,30–32^. When we introduce a passive screening layer [panels (c), (d)], we find qualitatively the same features. Quantitatively, however, the plasmon polaron replica features shift to lower energies, as seen in the frequency linecuts of *A*_**k**_(*ω*) at **k** = *Γ* [panels (e), (f)]. This is a consequence of the shift of plasmonic spectral weight to higher energies, due to the enhanced frequency of the in-phase plasmon mode and due to Landau damping of the out-of-phase plasmon mode.

### Plasmonic superconductivity

**Fig. 4:**
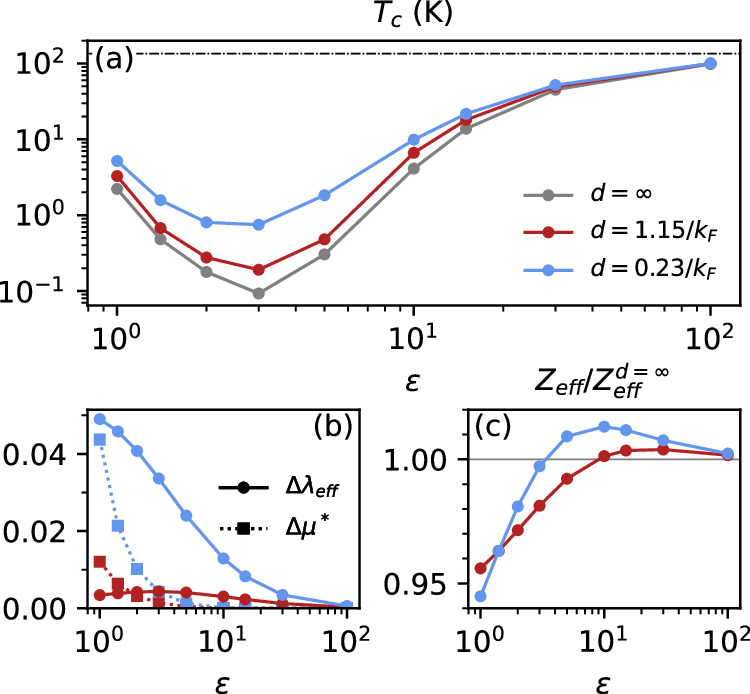
The superconducting critical temperature. **a** *T*_*c*_ within the active layer as a function of external screening *ε*, for different interlayer distances *d*. The horizontal dashed line denotes the *T*_*c*_ corresponding to *ε* = *∞*. **b** The change of the effective pairing strength $\Delta {\lambda }_{e\mathrm{ff}}={\lambda }_{e\mathrm{ff}}-{\lambda }_{e\mathrm{ff}}^{d=\infty }$ (solid lines) and of the TMA pseudo-potential $\Delta {\mu }^{* }={\mu }^{* }-{\mu }_{d=\infty }^{* }$ (dotted lines) upon introducing a metallic screening layer. **c** The relative change of the effective mass-renormalization factor ${Z}_{e\mathrm{ff}}/{Z}_{e\mathrm{ff}}^{d=\infty }$. These results were obtained at *T* = 100 K, with m^*^ = 0.2*m*_*e*_ and *E*_*F*_ = 1 eV in both layers.

Figure 4 (a) shows the superconducting critical temperatures *T*_*c*_ for different interlayer distances *d* as well as for different overall Coulomb interaction strengths tuned by *ε*. The infinite separation trace (gray line) reproduces previous results^15^. At *ε* = *∞* the only interactions contributing to superconductivity are the local LO and TO electron-phonon interactions and correspondingly we find negligible distance dependence of the critical temperature. As *ε* is decreased from *∞* to ~ 20 the calculated transition temperature decreases markedly because the high frequency Coulomb repulsion *U*_**q**_ increases as ∝ 1/*ε*, but the dynamical Coulomb interaction remains weak and thus the distance dependence of the transition temperature remains small. In this “phononic” regime environmental bosonic engineering is ineffective. As *ε* decreases further, *T*_*c*_ reaches a minimum around *ε* ≈ 3 while the dependence of the transition temperature on the interlayer distance becomes much stronger. Here the dominant effect is the continuing dramatic increase of the high frequency instantaneous Coulomb repulsion. However, the plasmonic pairing also begins to play a role, and may be engineered by the presence of the passive layer. Beyond this minimum, for *ε* ≲ 2, the plasmon mediated electron-electron attraction starts to dominate the interaction, leading again to an enhancement of *T*_*c*_. In this “plasmonic” regime the effect of the passive layer remains large.

### Qualitative description

To qualitatively disentangle the different contributions to the superconducting state, we use standard formulae (see “Methods” section) to approximate the full frequency dependent interaction in terms of an effective electron-electron coupling *λ*_eff_, an effective boson frequency *ω*_eff_, a Tolmachev-Morel-Anderson (TMA) Coulomb pseudo-potential μ* and a mass-renormalization factor *Z*_eff_ as used in the conventional discussions in phonon-mediated superconductors. With these parameters, we can calculate an effective critical temperature

3 $${k}_{B}{T}_{c}^{\mathrm{eff}}=1.13{\omega }_{\mathrm{eff}}\exp \left(-\frac{{Z}_{\mathrm{eff}}}{{\lambda }_{\mathrm{eff}}-{\mu }^{* }}\right).$$

This expression qualitatively reproduces the trends of our numerical results, but is quantitatively off. As in discussions of conventional superconductivity, this analysis should thus be understood as a tool to gain intuitive understanding but cannot be used for quantitative predictions.

In Fig. 4(b) we show that *λ*_eff_ increases with decreasing distance *d* as well as with decreasing *ε*. Thus, proximity to the screening layer enables a stronger effective electron-electron attraction in the active superconducting layer, which is induced by the additional pairing channel mediated by external dynamical boson modes. This is clearly a supporting mechanism of bosonic Coulomb engineering.

We have shown above that, as the interlayer distance is reduced, the spectral weight of the interaction shifts to higher bosonic energies as a consequence of the enhanced energy of the in-phase plasmon mode and the Landau-damping of the out-of-phase plasmon mode (see Fig. 2). The effective boson frequency *ω*_eff_ is therefore increased. On the one hand, this effect tends to enhance *T*_*c*_ due to *ω*_eff_ entering as a prefactor to the critical temperature. On the other hand, it also tends to reduce *T*_*c*_ by enhancing the TMA pseudo-potential μ*. Effects of changing *ω*_eff_ are mostly negligible in the phononic regime, but in the plasmonic regime *ε* ≲ 3 the enhanced *ω*_eff_ causes a significant enhancement of μ*. Nevertheless, for most values of *ε*, *λ*_eff_ is enhanced more than μ*. Somewhat paradoxically, it is therefore possible to tune the instantaneous repulsion *μ*^⋆^ via retardation effects, even though the bare Coulomb potential is not affected by the interlayer coupling. This dependence suggests an additional direction for bosonic engineering.

In Fig. 4(c) we further show the relative change of *Z*_eff_. Interestingly, the mass-renormalization is enhanced in the phononic regime, whereas it is reduced in the plasmonic regime. The actual changes are, however, only on the order of a few percent, such that mass-renormalization alone cannot explain the *T*_*c*_ enhancement. We therefore conclude that the driving force behind the enhancement of *T*_*c*_ is the enhancement of the effective electron-electron attraction *λ*_eff_, which is, however, weakened through an enhanced μ* in the low *ε* regime as a consequence of the enhanced *ω*_eff_. Our results thus show that bosonic Coulomb engineering via dynamical external screening can be utilized to strongly enhance plasmonic superconductivity in layered materials, leading to critical temperatures increased by factors of up to 20 as discussed in the following.

### Anomalous self-energy

**Fig. 5:**
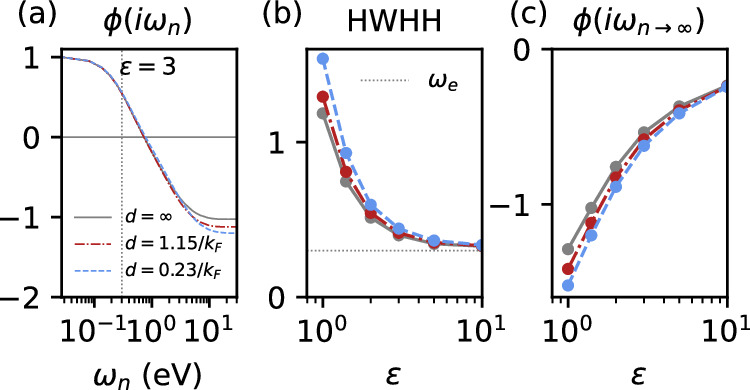
The anomalous self-energy. **a** The momentum-summed anomalous self-energy *ϕ*(*iω*_*n*_) in the active layer at *ε* = 3 as a function of Matsubara frequency *ω*_*n*_. The vertical dotted line indicates the bare phonon frequency *ω*_*e*_. **b** The half-width at half-height of *ϕ*(*iω*_*n*_). **c** The high-frequency tail of *ϕ*(*iω*_*n*_). These results were obtained at *T* = 100 K, with m* = 0.2*m*_*e*_ and *E*_*F*_ = 1 eV in both layers. In all cases *ϕ* is normalized such that *ϕ*(*iω*_*n*=0_) = 1.

In Fig. 5(a) we show the momentum-summed anomalous self-energy of the active layer *ϕ*(*iω*_*n*_) = ∑_**k**_*ϕ*(**k**, *iω*_*n*_) at *ε* = 3. Since *ϕ*(**k**, *iω*_*n*_) is obtained from the linearized gap equation, it has an arbitrary normalization factor. Here, the normalization factor was fixed such that *ϕ*(*iω*_*n*=0_) = 1. For all *d* we find the characteristic features of the crossover from phonon to plasmon mediated superconductivity as *ε* is reduced, as discussed in more detail for monolayers in previous work^15^. It has been shown that the frequency width of the anomalous self-energy, here illustrated using the half-width at half-height (HWHH), is proportional to a characteristic frequency of the mediating bosons^33–36^. For *ε* → *∞* we therefore find that the HWHH tends towards the bare phonon frequency *ω*_*e*_ [panel (b)], because all Coulombic contributions are suppressed in that regime. The increased half-width at half-height (HWHH) of *ϕ*(*iω*_*n*_) as *ε* reduces reflects that the dominating mediating boson switches from the lower-energy phonon mode to the higher-energy plasmon mode. Furthermore, the high-frequency tail [panel (c)] is reduced as *ε* reduces due to the enhanced high-frequency Coulomb repulsion at low *ε*.

Introducing the passive screening layer does not qualitatively change the trends of *ϕ*(*iω*_*n*_) as a function of *ε*, indicating that there is still a crossover from phonon to plasmon mediated superconductivity. Quantitatively there are some differences however. Firstly, the HWHH of *ϕ*(*iω*_*n*_) is enhanced by interlayer dynamical screening, which reflects that the spectral weight of the interaction is shifted to higher energies. This is consistent with the enhanced plasmon frequencies shown in Fig. 2 and with the enhanced *ω*_eff_. Secondly, the high-frequency tail of *ϕ*(*iω*_*n*_) is reduced upon reducing *d*. This can be analyzed in an approximate BCS picture, in which the high-frequency tail of *ϕ*(*iω*_*n*_) behaves as − μ*/(*λ*_eff_ − μ*)^33,36^. From the qualitative modeling above we expect the value of *λ*_eff_ to increase as the layers are brought closer together. The reduced high-frequency tail therefore indicates that the pseudopotential μ* is also enhanced upon reduced *d*. These results agree with our qualitative modeling and again hint towards a delicate balance between dynamical attraction, and (renormalized) instantaneous repulsion.

### Optimizing superconductivity via bosonic engineering

To enhance *T*_*c*_ the screening layer should lead to a low-energy composite bosonic mode, which couples strongly to the electrons. This could be achieved by tuning the electronic properties of the screening layer. In our model this is controlled by its effective mass as well as by its doping level. While the carrier concentration in the passive layer can be controlled by gating, the effective mass is difficult to control in situ. However, passive layers could be chosen to have heavy mass carriers.

**Fig. 6:**
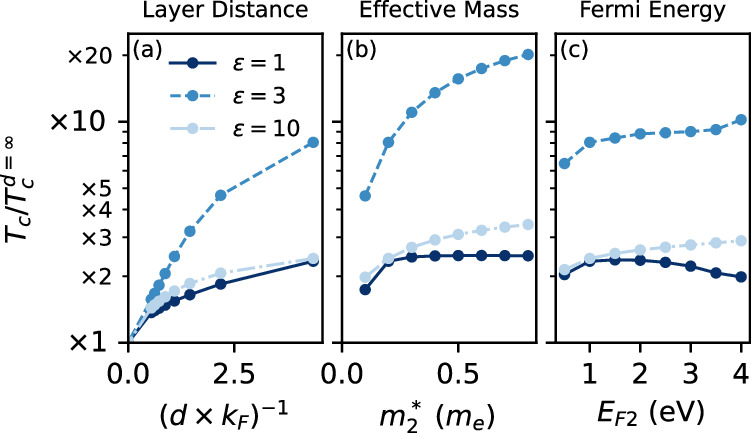
Enhancement of the critical temperature. The enhancement of the superconducting critical temperature with respect to an isolated monolayer ${T}_{c}/{T}_{c}^{d=\infty }$, as a function of the inverse interlayer distance (**a**), the effective mass of the screening layer (**b**) and the Fermi energy of the screening layer (**c**). We use as a baseline *d* × *k*_*F*_ = 0.23, ${m}_{2}^{* }=0.2{m}_{e}$ and *E*_*F*2_ = 1 eV and vary the respective parameters from there.

In Fig. 6 we show the relative change of the full numerically evaluated *T*_*c*_ due to the presence of the passive layer, as a function of different tuning parameters. In panel (a) we again find a significant *T*_*c*_ enhancement as the interlayer distance is reduced. Especially at *ε* = 3 this enhancement is a factor of 8 for the smallest *d* considered, but also for *ε* = 1 and *ε* = 10 we find up to a doubling of the critical temperature. In Fig. 6(b) we show the results when tuning the effective mass of the passive layer ${m}_{2}^{* }$ at fixed interlayer distance *d* × *k*_*F*_ = 0.23. In these calculations the total electron density was kept fixed by adjusting the Fermi energy *E*_*F*2_ correspondingly.

**Fig. 7:**
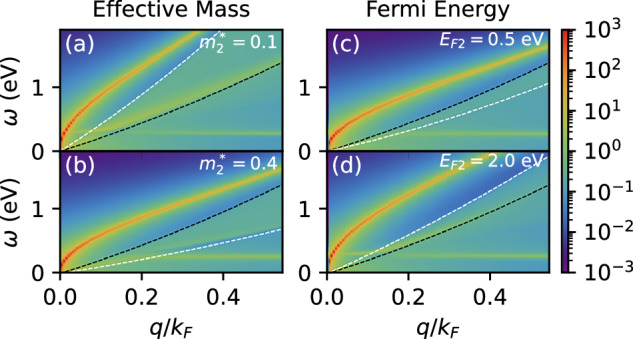
Tunability of the effective interaction. The effective interaction $2{\alpha }^{2}{F}_{{\bf{q}}}^{L}(\omega )/\omega$ within the active layer, at *d* × *k*_*F*_ = 0.23 and *ε* = 3, upon tuning the effective mass (**a**, **b**) and the Fermi energy (**c**,**d**) of the passive layer. We use as a baseline ${m}_{2}^{* }=0.2{m}_{e}$ and *E*_*F*2_ = 1 eV and vary the respective parameters from there. The dashed black (white) lines denote the upper edge of the electron-hole continuum of the active (passive) layer.

As we show in Fig. 7(a), (b), enhancing ${m}_{2}^{* }$ pushes all plasmon modes to lower energies, which leads to a reduced *ω*_eff_ and thus a reduced μ*. At the same time, the edge of the electron-hole continuum of the screening layer (boundary indicated by the white dashed lines) is also shifted to lower energies, which suppresses Landau damping and thus leads to an enhanced *λ*_eff_. This interplay of enhancing *λ*_eff_ and reducing μ* is optimal to increase *T*_*c*_, leading to the strong enhancement in Fig. 6(b). Overall we conclude that a metallic screening layer with a large effective mass is most favorable for enhancing plasmonic pairing in a layered superconductor.

In Fig. 6(c), we summarize the effect of tuning the Fermi energy of the screening layer *E*_*F*2_ at fixed effective mass ${m}_{2}^{* }$, thereby changing the electron density in the screening layer. Enhancing *E*_*F*2_ causes the in-phase plasmon mode to shift to higher energies, as shown in Fig. 7(c), (d). This leads to an enhancement of μ*, while *λ*_eff_ is relatively unaffected, such that the combined Δ*λ*_eff_ − Δμ* suggests a reduction of the critical temperature with increased doping. Since *Z*_eff_ is reduced as well, *T*_*c*_ is ultimately relatively unaffected by modifications to the doping in the screening layer. Interestingly, for *ε* = 1 we find a dome-like shape as a function of *E*_*F*2_, which is reminiscent of earlier works by Fatemi and Ruhman^18^ and by Takada^37^. Overall, we see a strong, but not doping-tunable *T*_*c*_ enhancement as a result of the presence of the screening layer.

## Discussion

We have shown that in all cases considered, bosonic engineering of plasmonic modes via interlayer dynamical screening can enhance the superconducting critical temperature of two-dimensional materials. Especially in the regime of intermediate Coulomb interaction strengths, where in the monolayer limit *T*_*c*_ has a minimum due to the unfavorable interplay of static Coulomb repulsion and dynamic pairing, *T*_*c*_ can be enhanced by more than an order of magnitude. The driving mechanism behind this enhancement is the formation of composite interlayer plasmon modes, which have a total coupling strength to the electrons that is larger than that of the individual monolayer plasmon modes. The enhanced coupling strength is however counteracted by changes in the Coulomb pseudopotential μ* and the mass-renormalization *Z*_eff_, which are also affected by dynamical interlayer screening. Our results show that, in order to get the most favorable combination of all these competing effects for high *T*_*c*_, the electrons in the screening layer should have large effective mass. We furthermore find that the electron density of the screening layer is significantly less relevant than the effective mass for engineering optimal *T*_*c*_.

Beyond the implications for bosonic engineering, our work shows that appropriately designed structures can provide evidence relevant to the long-standing question of plasmon mediated (or enhanced) superconductivity. The balance between static repulsion and dynamical attraction defines *T*_*c*_ and one would like to know to which extent dynamical screening associated with plasmons can lead to pairing. We find that proximity to a passive layer can enhance superconductivity in the plasmonic regime, but not in the phononic regime; thus observation of increases in *T*_*c*_ due to the presence of a passive layer indicates that plasmonic pairing is in play. To this end, the electron-doped semiconducting transition metal dichalcogenides (TMDCs) are especially promising. Some of the TMDCs, such as MoS_2_^38^ and WS_2_^39^, have been shown to superconduct in the monolayer limit upon electron doping. Moreover, the normal state of TMDCs is known to be sensitive to (dynamical) environmental screening^3,5^ and the conduction band minimum is well described by an effective mass approximation^40,41^. Heterostructures of such a superconducting TMDC monolayer and another metallic monolayer with larger effective mass, such as NbTe_2_^40,41^, separated by a hBN monolayer might therefore be an experimental realization of the model discussed here.

Our results could furthermore be relevant in the observed *T*_*c*_ enhancement in electron-doped semi-conducting TMDC multi-layers as the number of layers is increased^42–44^. Previous work has shown that effects from static screening alone cannot explain this behavior^45^, but additional pairing strength from interlayer plasmon modes might explain the *T*_*c*_ enhancement.

Finally, bosonic engineering is not restricted to plasmon modes alone. Other bosons in the environment might similarly form interlayer composite boson modes whenever their dispersions overlap with boson dispersions in the active layer and whenever their excitation energies lie outside the regime of Landau damping. This might be relevant for the drastic *T*_*c*_ enhancement in monolayer FeSe on SrTiO_3_, which has been argued to be induced by SrTiO_3_ phonon modes coupled into the FeSe layer^46,47^. From our results we hypothesize that this enhancement may instead result from the renormalization of FeSe boson modes (e.g., phonons, plasmons, or magnons) due to hybridization with external SrTiO_3_ bosonic modes, and not from pairing induced by SrTiO_3_ phonons on their own.

## Methods

### Model

We study a hetero-bilayer system consisting of two layers which are electronically decoupled. The electronic structure of both layers is described by an effective-mass approximation ${\varepsilon }_{{\bf{k}},i}={k}^{2}/(2{m}_{i}^{* })-{E}_{F,i}$, with ${m}_{i}^{* }$ and *E*_*F*,*i*_ the effective mass and Fermi energy in layer *i*, respectively, where *i* = 1 is the active superconducting layer and *i* = 2 is the passive screening layer. We consider the following Hamiltonian

4 $$\begin{array}{cl}H & =\mathop{\sum }\limits_{{\bf{k}}i\sigma }{\varepsilon }_{{\bf{k}},i}{c}_{{\bf{k}}i}^{\sigma ,\dagger }{c}_{{\bf{k}}i}^{\sigma }+\frac{1}{2}\mathop{\sum }\limits_{{{\bf{kk}}}^{{\prime} }{\bf{q}}}\mathop{\sum }\limits_{ij\sigma {\sigma }^{{\prime} }}{\widehat{U}}_{ij}({\bf{q}}){c}_{{\bf{k}}+{\bf{q}}i}^{\sigma ,\dagger }{c}_{{{\bf{k}}}^{{\prime} }-{\bf{q}}\,j}^{{\sigma }^{{\prime} },\dagger }{c}_{{{\bf{k}}}^{{\prime} }j}^{{\sigma }^{{\prime} }}{c}_{{\bf{k}}i}^{\sigma }\\ & +\mathop{\sum }\limits_{{\bf{q}}\nu }{\omega }_{e}{b}_{{\bf{q}}\nu }^{\dagger }{b}_{{\bf{q}}\nu }+\mathop{\sum }\limits_{{\bf{kq}}\nu \sigma }g({b}_{{\bf{q}}\nu }+{b}_{-{\bf{q}}\nu }^{\dagger }){c}_{{\bf{k}}+{\bf{q}}1}^{\sigma ,\dagger }{c}_{{\bf{k}}1}^{\sigma },\end{array}$$

where ${c}_{{\bf{k}}i}^{\sigma ,(\dagger )}$ is the annihilation (creation) operator of electrons in layer *i* with momentum **k** and spin *σ*. ${b}_{{\bf{q}}\nu }^{(\dagger )}$ is the annihilation (creation) operator of phonons with transfer momentum **q**, which we include in the active superconducting layer only. Here *ν* ∈ {LO, TO} for the longitudinal optical (LO) and transverse optical (TO) phonon mode, respectively, *g* is the electron-phonon coupling constant and *ω*_*e*_ is the phonon energy, which we both assume to be local for simplicity. The matrix elements of the long-range Coulomb interaction in the density-density approximation are given in the layer-basis by

5 $$\widehat{U}({\bf{q}})={U}_{{\bf{q}}}\left(\begin{array}{cc}1 & {e}^{-qd}\\ {e}^{-qd} & 1\end{array}\right),$$

where *U*_**q**_ = 2*πe*^2^/(*Aεq*), with *A* the unit-cell area, *d* the distance between the two layers and *ε* a global dielectric constant. In this basis the diagonal and off-diagonal components represent intra- and interlayer interactions, respectively.

Following usual many-body perturbation theory methods, we find that the longitudinal part of the total screened interaction matrix in the RPA is given by a Dyson equation

6 $${\widehat{I}}^{\,L}({\bf{q}},i{\nu }_{m})={\widehat{I}}^{\,(0)}({\bf{q}},i{\nu }_{m})+{\widehat{I}}^{\,(0)}({\bf{q}},i{\nu }_{m}){\widehat{\Pi }}^{(0)}({\bf{q}},i{\nu }_{m}){\widehat{I}}^{\,L}({\bf{q}},i{\nu }_{m}).$$

Here the bare interaction is obtained by integrating out the phonons in the active layer yielding ${\widehat{I}}^{\,(0)}({\bf{q}},i{\nu }_{m})=\widehat{U}({\bf{q}})+{\widehat{I}}_{ph}^{\,(0),LO}({\bf{q}},i{\nu }_{m})$^8^, where

7 $${\widehat{I}}_{ph}^{\,(0),LO/TO}({\bf{q}},i{\nu }_{m})=\left(\begin{array}{cc}{I}_{ph}^{(0),TO/LO}(i{\nu }_{m}) & 0\\ 0 & 0\end{array}\right)$$

and ${I}_{ph}^{(0),LO/TO}(i{\nu }_{m})=2{\omega }_{e}{g}^{2}/({(i{\nu }_{m})}^{2}-{\omega }_{e}^{2})$. The RPA polarization matrix ${\widehat{\Pi }}^{(0)}({\bf{q}},i{\nu }_{m})$ is diagonal as a consequence of the vanishing interlayer electronic hybridization

8 $${\widehat{\Pi }}^{(0)}({\bf{q}},i{\nu }_{m})=\left(\begin{array}{cc}{\Pi }_{1}^{(0)}({\bf{q}},i{\nu }_{m}) & 0\\ 0 & {\Pi }_{2}^{(0)}({\bf{q}},i{\nu }_{m})\end{array}\right),$$

where ${\Pi }_{i}^{(0)}({\bf{q}},i{\nu }_{m})$ is the Lindhard function evaluated in layer *i*. The TO phonon mode is assumed to be unscreened in the RPA, such that the total interaction is given by $\widehat{I}({\bf{q}},i{\nu }_{m})={\widehat{I}}^{\,L}({\bf{q}},i{\nu }_{m})+{\widehat{I}}_{ph}^{(0),TO}(i{\nu }_{m})$. Inverting Eq. (6) finally yields for the screened interaction

9 $$\begin{array}{ll}\widehat{I}({\bf{q}},i{\nu }_{m}) & ={\left[\widehat{{\mathcal{I}}}-(\widehat{U}({\bf{q}})+{\widehat{I}}_{ph}^{\,(0),LO}({\bf{q}},i{\nu }_{m})){\widehat{\Pi }}^{(0)}({\bf{q}},i{\nu }_{m})\right]}^{-1}\\ & \times (\widehat{U}({\bf{q}})+{\widehat{I}}_{ph}^{\,(0),LO}({\bf{q}},i{\nu }_{m}))\\ & +{\widehat{I}}_{ph}^{\,(0),TO}(i{\nu }_{m}).\end{array}$$

We note that the poles of Eq. (9) are equivalent to those obtained in classical works on plasmons in bilayer systems, if the phonons are neglected^24,25^.

From here, we derive an effective single layer model. Because we neglect interlayer electronic hybridization and because the interactions are treated in the density-density approximation, the G_0_W_0_ self-energy is diagonal in our model. The component within the active layer is given by

10 $$\Sigma ({\bf{k}},i{\omega }_{n})=-\frac{1}{\beta }\mathop{\sum }\limits_{{{\bf{k}}}^{{\prime} },{n}^{{\prime} }}{I}_{1}({\bf{k}}-{{\bf{k}}}^{{\prime} },i{\omega }_{n}-i{\omega }_{{n}^{{\prime} }}){G}_{1}^{(0)}({{\bf{k}}}^{{\prime} },i{\omega }_{{n}^{{\prime} }}),$$

where ${G}_{1}^{(0)}$ is the bare Green’s function in the active layer. Similarly, we neglect inter-layer Cooper pairing, which also results in a diagonal anomalous self-energy. The component within the active layer is

11 $$\begin{array}{ll}\phi ({\bf{k}},i{\omega }_{n}) & =-\frac{1}{\beta }\mathop{\sum }\limits_{{{\bf{k}}}^{{\prime} },i{\omega }_{{n}^{{\prime} }}}{I}_{1}({\bf{k}}-{{\bf{k}}}^{{\prime} },i{\omega }_{n}-i{\omega }_{{n}^{{\prime} }})\\ & \times {G}_{1}^{(0)}({{\bf{k}}}^{{\prime} },i{\omega }_{{n}^{{\prime} }})\phi ({{\bf{k}}}^{{\prime} },i{\omega }_{{n}^{{\prime} }}){G}_{1}^{(0)}(-{{\bf{k}}}^{{\prime} },-i{\omega }_{{n}^{{\prime} }}).\end{array}$$

Both the normal and anomalous self-energies only depend on the component of the screened interaction matrix corresponding to the active layer *I*_1_, which means we only need to evaluate this component from Eq. (9). This yields an effective single-layer model with a renormalized screened interaction given by ${I}_{1}({\bf{q}},i{\nu }_{m})={I}_{1}^{L}({\bf{q}},i{\nu }_{m})+{I}_{ph}^{(0),TO}(i{\nu }_{m})$. We find that the longitudinal part ${I}_{1}^{L}({\bf{q}},i{\nu }_{m})$ is given by

12 $${I}_{1}^{L}({\bf{q}},i{\nu }_{m})=\frac{{\widetilde{I}}_{1}^{\,(0),L}({\bf{q}},i{\nu }_{m})}{1-{\widetilde{I}}_{1}^{\,(0),L}({\bf{q}},i{\nu }_{m}){\Pi }_{1}^{(0)}({\bf{q}},i{\nu }_{m})},$$

with the effective interaction ${\widetilde{I}}_{1}^{(0),L}({\bf{q}},i{\nu }_{m})$ given by Eq. (2) and ${\Pi }_{1}^{(0)}({\bf{q}},i{\nu }_{m})$ the RPA polarization in the active superconducting layer. We fix for the active layer ${m}_{1}^{* }=0.2\,{m}_{e}$ and *E*_*F*1_ = 1 eV, while tuning ${m}_{2}^{* }$ and *E*_*F*2_ of the passive layer. Here *m*_*e*_ is the free electron mass. For both the LO and TO bare phonon modes we set *ω*_*e*_ = 0.3 eV and *g*^2^ = 0.3 eV^2^.

### Computational details

Calculations were performed using the TRIQS^48^ and TPRF^49^ codebases, using a linearly discretized momentum mesh of 800 × 800 points. The Matsubara axis was represented using the recently developed discrete Lehman representation (DLR)^50^, which drastically reduces the temperature scaling of the required amount of Matsubara frequencies to ${\mathcal{O}}(\log (\beta ))$ compared to a full Matsubara mesh which scales as ${\mathcal{O}}(\beta )$. Due to the improved scaling with temperature, we can resolve the superconducting critical temperatures in the low screening limit. For all calculations, the DLR error tolerance was set to *ϵ* = 10^−10^ and the high-energy cutoff to ${\omega }_{\max }=50$ eV. Critical temperatures were obtained by evaluating the leading eigenvalue *λ*(*T*) of the linearized gap equation at logarithmically spaced temperatures between 1 K and 100 K. We then perform a linear fit of *λ*(*T*) as a function of $\log (T)$, which yields *T*_*c*_ as the temperature at which *λ*(*T*) = 1. Real-frequency G_0_W_0_ calculations have been performed on a 100 × 100 momentum mesh and a linearly spaced frequency mesh with 1000 points between −20 and 20 eV.

### Qualitative modeling

Our qualitative modeling of the different contributions to the superconducting state closely follows the McMillan-Allen-Dynes description for phonon-mediated superconductors^51–54^. We define the following expression for the effective critical temperature

13 $${k}_{B}{T}_{c}^{\mathrm{eff}}=1.13{\omega }_{\mathrm{eff}}\exp \left(-\frac{{Z}_{\mathrm{eff}}}{{\lambda }_{\mathrm{eff}}-{\mu }^{* }}\right).$$

Here the effective dimensionless electron-electron pairing strength *λ*_eff_ is defined as the momentum integral of the zero-frequency electron-electron coupling

14 $$\begin{array}{ll}{\lambda }_{\mathrm{eff}} & =-{N}_{0}\mathop{\sum }\limits_{{\bf{q}}}({I}_{1}({\bf{q}},i{\nu }_{m=0})-{U}_{{\bf{q}}})\\ & ={\int }_{0}^{\infty }d\omega \mathop{\sum }\limits_{{\bf{q}}}\frac{2{\alpha }^{2}{F}_{{\bf{q}}}^{L}(\omega )}{\omega }+\frac{2{g}^{2}{N}_{0}}{{\omega }_{e}},\end{array}$$

where *N*_0_ is the density of states at the Fermi energy. This effective coupling is counteracted by the static Coulomb repulsion. Similar to the coupling, we will define a corresponding dimensionless parameter *μ*^*C*^. A common definition of *μ*^*C*^ is a double Fermi surface average of *U*_**q**_.^33,55,56^ Here, we instead define it by the momentum sum *μ*^*C*^ = *N*_0_∑_**q**_*U*_**q**_ for consistency with *λ*_eff_. From Eliashberg theory we understand, however, that it is not the bare potential *μ*^*C*^ that enters the effective low-energy gap-equation, but the renormalized TMA pseudo-potential μ*^33,57^, given by

15 $${\mu }^{* }=\frac{{\mu }^{C}}{1+{\mu }^{C}\log ({E}_{B}/{\omega }_{\mathrm{eff}})},$$

where *E*_*B*_ is the bandwidth (set here to *E*_*B*_ = 4*E*_*F*1_ for simplicity). The effective boson frequency *ω*_eff_ we define using the logarithmic average

16 $${\omega }_{\mathrm{eff}} =\exp \left[\frac{2}{{\lambda }_{\mathrm{eff}}}{\int }_{0}^{\infty }d\omega \mathop{\sum }\limits_{{\bf{q}}}{\alpha }^{2}{F}_{{\bf{q}}}^{L}(\omega )\frac{\log (\omega )}{\omega }\right] \exp \left[\frac{2}{{\lambda }_{\mathrm{eff}}}\frac{{g}^{2}{N}_{0}\log ({\omega }_{e})}{{\omega }_{e}}\right].$$

The final contribution to the critical temperature is the mass-renormalization factor *Z*_eff_. Similar to Eliashberg theory, we define it using the frequency derivative of the G_0_W_0_ self-energy evaluated at *k*_*F*_

17 $${Z}_{\mathrm{eff}}=1-\left.{{\frac{\partial \Sigma ({k}_{F},i{\omega }_{n})}{\partial (i{\omega }_{n})}}}\right|_{i{\omega }_{n}=0}.$$

## Data Availability

All relevant data needed to reproduce our results can be found at 10.34973/pcfv-0t58.

## Code availability

All calculations were performed using the TRIQS and TPRF software libraries, which are freely available at https://triqs.github.io/triqs and https://triqs.github.io/tprf. Additional code and plotting scripts can be found at 10.34973/pcfv-0t58.
